# Systems Biological Approach of Molecular Descriptors Connectivity: Optimal Descriptors for Oral Bioavailability Prediction

**DOI:** 10.1371/journal.pone.0040654

**Published:** 2012-07-16

**Authors:** Shiek S. S. J. Ahmed, V. Ramakrishnan

**Affiliations:** 1 Department of Computational Biology, Chettinad University, Kelambakkam, Tamil Nadu, India; 2 Department of Genetics, Chettinad University, Kelambakkam, Tamil Nadu, India; Aligarh Muslim University, India

## Abstract

**Background:**

Poor oral bioavailability is an important parameter accounting for the failure of the drug candidates. Approximately, 50% of developing drugs fail because of unfavorable oral bioavailability. *In silico* prediction of oral bioavailability (%F) based on physiochemical properties are highly needed. Although many computational models have been developed to predict oral bioavailability, their accuracy remains low with a significant number of false positives. In this study, we present an oral bioavailability model based on systems biological approach, using a machine learning algorithm coupled with an optimal discriminative set of physiochemical properties.

**Results:**

The models were developed based on computationally derived 247 physicochemical descriptors from 2279 molecules, among which 969, 605 and 705 molecules were corresponds to oral bioavailability, intestinal absorption (HIA) and caco-2 permeability data set, respectively. The partial least squares discriminate analysis showed 49 descriptors of HIA and 50 descriptors of caco-2 are the major contributing descriptors in classifying into groups. Of these descriptors, 47 descriptors were commonly associated to HIA and caco-2, which suggests to play a vital role in classifying oral bioavailability. To determine the best machine learning algorithm, 21 classifiers were compared using a bioavailability data set of 969 molecules with 47 descriptors. Each molecule in the data set was represented by a set of 47 physiochemical properties with the functional relevance labeled as (+bioavailability/−bioavailability) to indicate good-bioavailability/poor-bioavailability molecules. The best-performing algorithm was the logistic algorithm. The correlation based feature selection (CFS) algorithm was implemented, which confirms that these 47 descriptors are the fundamental descriptors for oral bioavailability prediction.

**Conclusion:**

The logistic algorithm with 47 selected descriptors correctly predicted the oral bioavailability, with a predictive accuracy of more than 71%. Overall, the method captures the fundamental molecular descriptors, that can be used as an entity to facilitate prediction of oral bioavailability.

## Introduction

Systems biology is an emerging field that uses molecular connectivity approach to understand the biological phenomena on a wide scale. This approach of network reconstruction has proven successful in determining the disease mechanisms, drug targets and biomarkers for various diseases [1]–[3]. Similar approach is adopted in this study to integrate the physicochemical properties of the molecules, to determine the major contributing factors associated with oral bioavailability prediction. This ultimately provides optimal descriptors for predicting a potent pharmaceutical agent with improved absorption, distribution, metabolism, and excretion (ADME) properties.

ADME plays a crucial role in determining the pharmacokinetics of a drug candidate and thus its therapeutic efficacy [4]. Structural optimization of drug candidates with ADME properties has become an essential part of the drug discovery process [5]. Every successful drug candidate should ensure to achieve an optimal degree of potency with required concentration against specific the target. However, inadequate properties of the drug candidates will be failed while advanced development. It is believed that 50% of the drug candidates failed due to ADME deficiencies during development [6], [7]. Among the ADME properties, poor oral bioavailability is indeed the main reason for stopping further development of the drug candidates [8]. To overcome the failure, a set of *in vivo* screening has been carried out, to select the best oral bioavailability compounds at an early stage of the drug discovery process [9]. However, *in vivo* validations are expensive and time-consuming. Hence, developing an efficient *in silco* model for oral bioavailability screening will be more valuable [10].

**Figure 1:**
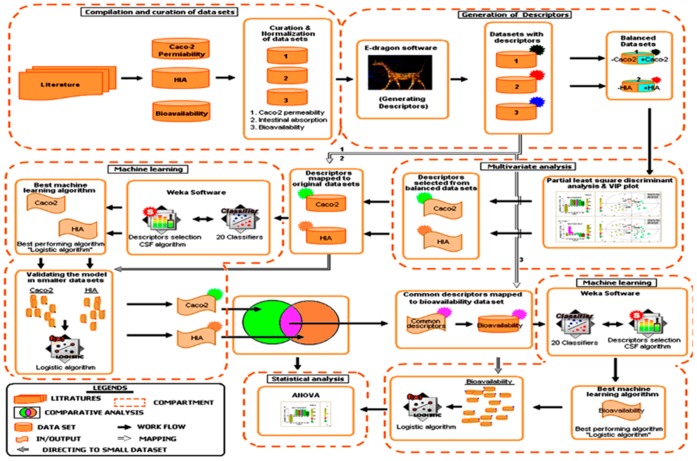
Systems biological framework for developing molecular descriptors connectivity maps. The framework consists of five major components: (i) compilation and curation of data sets, (ii) generation of descriptors (iii) multivariate analysis (iv) machine learning and (v) statistical analysis. The first component takes the inputs from literature and outputs the curated data sets. The second component takes the input from the curated data sets and generates molecular descriptors using E-dragon software. In the third component, HIA and caco-2 permeability data sets were subjected to multivariate analysis to obtain the most contributing descriptors involved in classification of groups against each data set. In the fourth component, the contributing descriptors were subjected to machine learning approach to determine the predictive accuracy of the models. The final statistical component was generated for the descriptors associated between data sets showing the interdependence between the descriptors.

In recent years, research efforts have resulted in the prediction of oral bioavailability of molecules [8], [11]–[17]. Most of these models were conceptually based on quantitative-structure activity relationship approach (QSAR) to study the physicochemical properties of molecules on oral bioavailability prediction. The first correlation model was reported based on a data set of 608 compounds [11]. This model is not satisfactory because of the high rate of false positives. Subsequently, Yoshida *et al* proposed a classification model based on 232 compounds with 18 descriptors that showed 60% accuracy [12]. Similarly, Turner *et al* built a stepwise regression model with 167 compounds using eight molecular descriptors [13]. Together, the models reported by Yoshida and Turner used smaller data sets. Hence, the reliability of these models is questionable in larger data set. In 2002, Veber *et al* demonstrated a QSAR model using 1100 drug candidates [14]. Further, Wang *et al* proposed a correlation model for 577 compounds using 50 descriptors [15]. Both these models utilized the drug data obtained from drug companies, hence it is access protected. In 2008, Ma *et al* developed a classification model using simple vector machine, which achieved 80% success rate [16]. However, this model could not give reliable accuracy for the low-bioavailability class. Considering the unbalanced nature of the data set, a prediction accuracy of 80% is meaningless since the model cannot provide better predictions for the low-bioavailability class. Hence developing a model with the balanced data set and including the parameters that influence the oral bioavailability such as HIA and caco-2 may provide reasonable prediction. On the other hand, these models were focused to obtain better accuracy with the simple molecular descriptors for oral bioavailability. However, recent studies of Hou *et al* and Tian *et al* disproved this concept of oral bioavailability prediction [8], [17]. Overall, no model gives reliable predictions for oral bioavailability. Moreover, the prediction of oral bioavailability is challenging due to the fact that oral bioavailability of the drug is influenced by many biological and physiochemical factors such as intestinal absorption, permeability, solubility, metabolism, and so forth [10], [18], [19]. Among the various factors intestinal absorption and permeability are the most contributing factors that influence oral bioavailability. Hence, we hypothesized that, the molecular properties associated intestinal absorption and permeability may have a high contribution in predicting the oral bioavailability.

**Figure 2:**
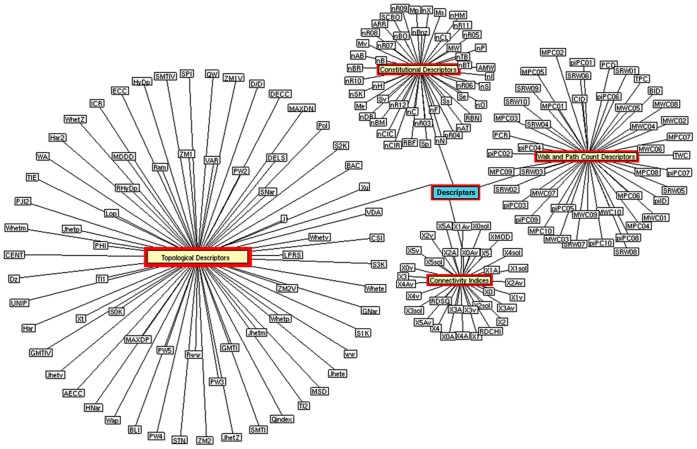
Analyzed descriptors. Four major descriptors (red) and their respective descriptor sub-classes were analyzed in this study.

In this study, we propose a computational framework to develop a molecular descriptors connectivity to uncover the most contributing descriptors for oral bioavailability by relating the intestinal absorption and permeability of the molecules in the physiochemical context. Overall, the potential application of our approach is to identify the optimal descriptors for oral bioavailability prediction with increased accuracy.

**Figure 3:**
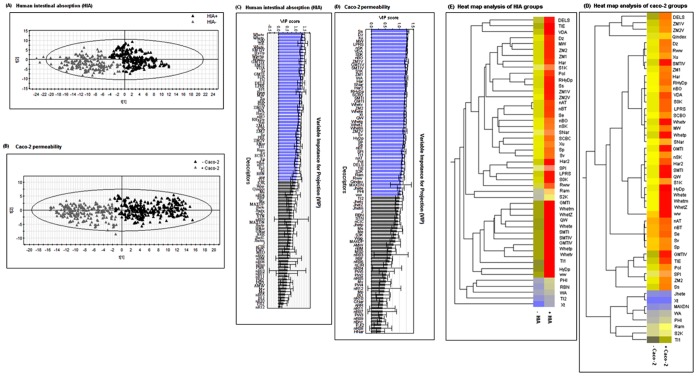
Multivariate analysis. PLS-DA plots: HIA (panel A) and caco-2 permeability (panel B) showing a significant differentiation (*p≤0.01* by permutation test) between the groups. The observations were coded according to class membership: black  =  positive; gray  =  negative. The descriptors which have a VIP score ≥1 were selected (colored blue) as the most contributing descriptors for HIA (panel C) and caco-2 (panel D). Heat map analysis of descriptors between positive and negative instance of HIA (panel E) and caco-2 (panel F) which depicts high (red) and low (yellow) relative levels of descriptor variations.

**Figure 4:**
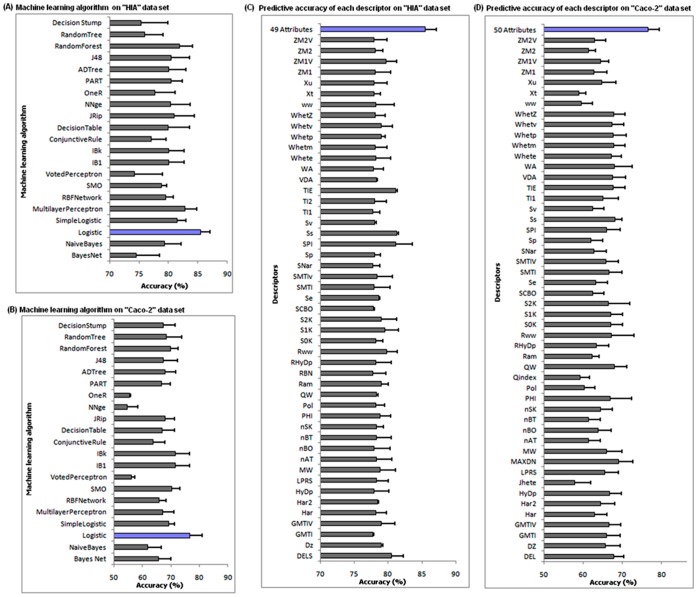
Machine learning algorithm. The performance of 21 machine learning algorithms for the prediction of HIA (panel A) and caco-2 (panel B) data sets were measured as averaged accuracy of 10-fold cross-validation analysis (the algorithm showing highest predictive accuracy indicated in blue). The predictive accuracy of the logistic algorithm was based on individual descriptors compared with the combined descriptors of HIA (panel C) and caco-2 data sets (panel D).

**Figure 5:**
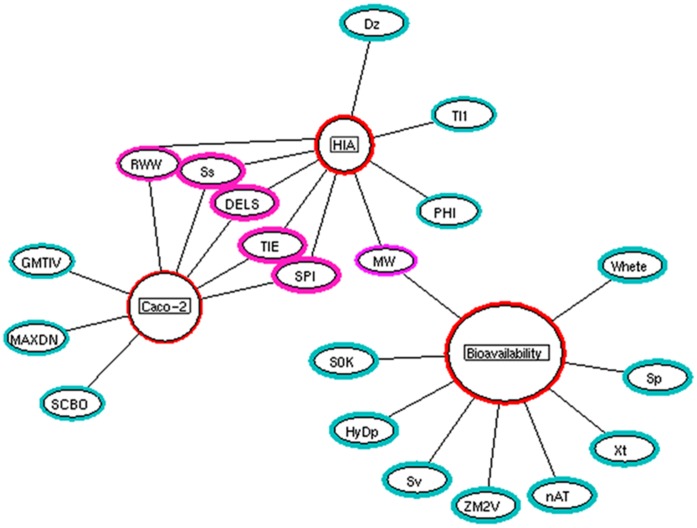
Correlation-based feature selection (CFS). The network representing the descriptors obtained using CFS algorithm showing common (pink) and unique (blue) descriptors between the data sets.

**Figure 6:**
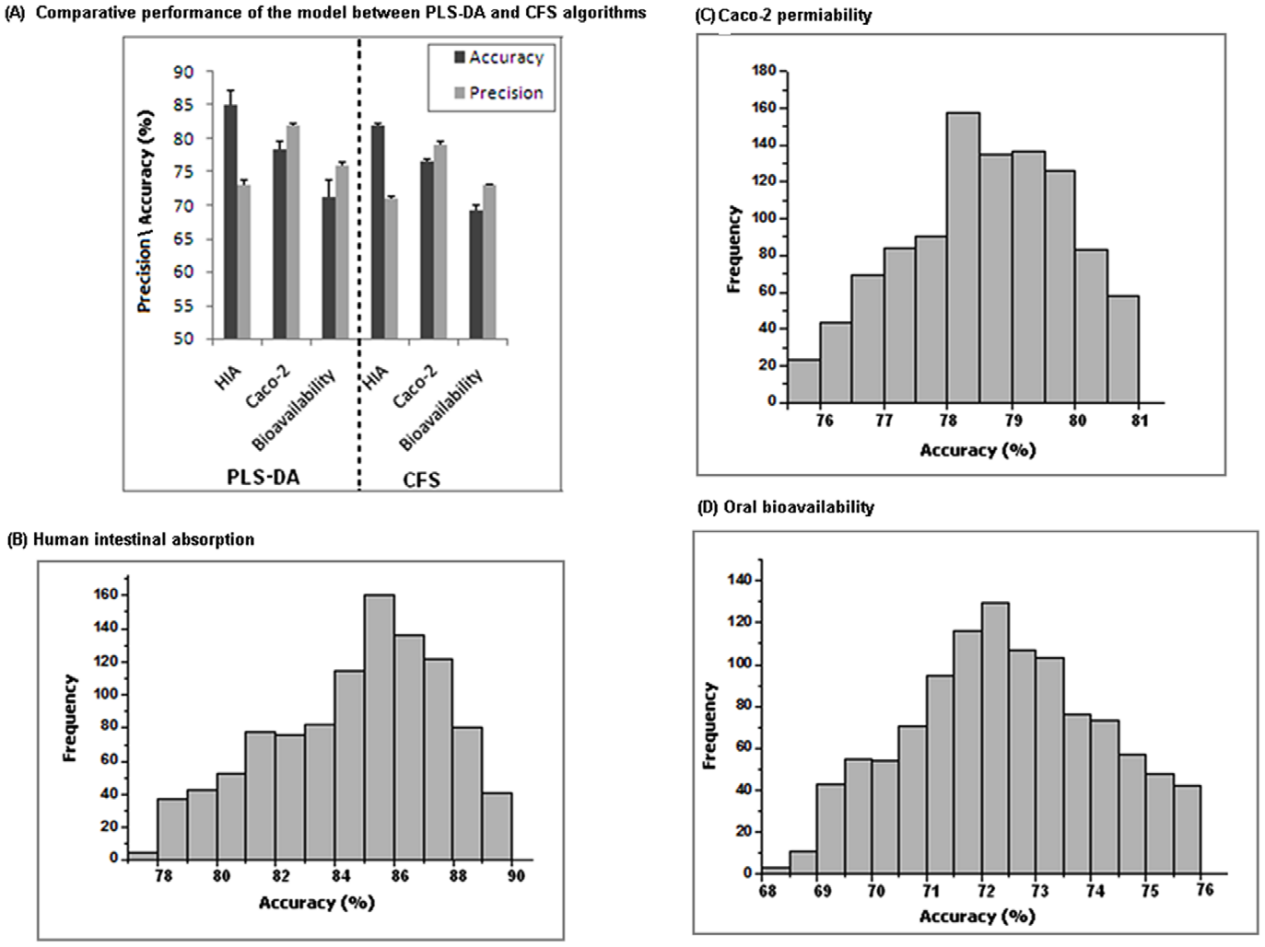
Comparative performance of the logistic model. Bar diagram (panel A) representing the comparative performance of the logistic model for the descriptors selected using PLS-DA and CFS algorithms. The histogram (panel B, C and D) shows the accuracy distribution of smaller data sets of HIA (***m  = 681***)**,** caco-2 (***m  = 710***) and oral bioavailability (***m  = 741***).

**Figure 7:**
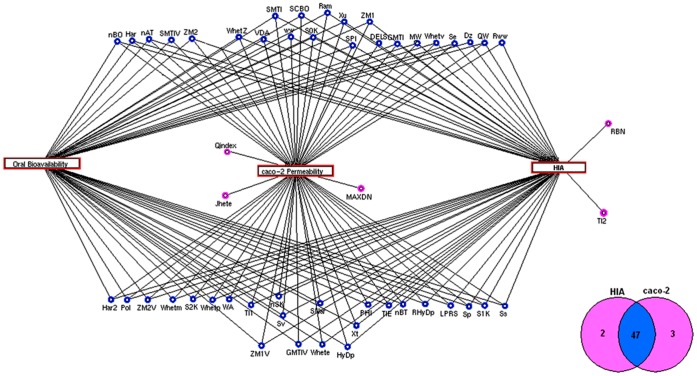
Descriptors interaction analysis. Descriptors interaction map showing the unique (pink) and common (blue) descriptors between HIA and caco-2 data sets. The commonly associated 47 descriptors (blue) were considered as the most contributing descriptors for the oral bioavailability prediction.

**Figure 8:**
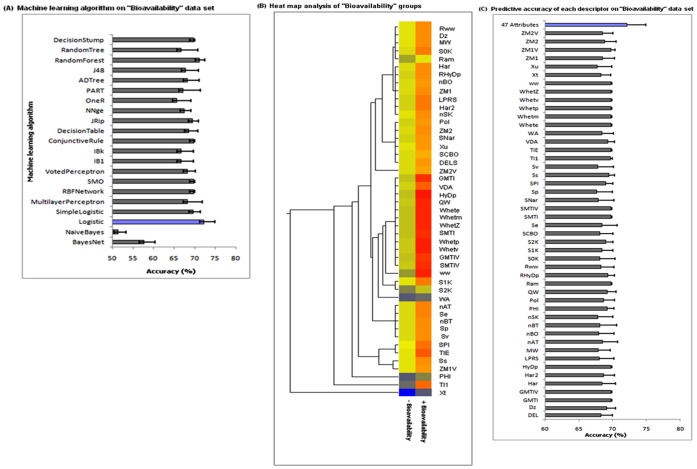
Oral bioavailability models. The performance of machine learning algorithms for the prediction of oral bioavailability (panel A) was measured as the average accuracy of 10-fold cross-validation (the algorithm showing highest predictive accuracy indicated in blue). Heat map analysis of descriptors between positive and negative instance of oral bioavailability (panel B) showing significant difference in descriptors depicts the high (red) and low (yellow) relative levels of descriptor variations. The predictive accuracy of the logistic algorithm was based on individual descriptors compared to the 47 combined descriptors of oral bioavailability data set (panel C).

## Results and Discussion

We have developed systems biological framework (Figure 1) of molecular descriptor connectivity between HIA and caco-2 permeability of diverse molecules obtained from the literature [8], [14], [20]–[28]. Each curated data set containing 605 molecules of HIA and 705 molecules of caco-2 was used as *original data sets* (see Table S1) for further analyses. E-dragon software was used to generate the connectivity indices, constitutional, topological, and walk and path counts descriptors against each data set based on the previous studies [29]–[32]. For instance, Gayathri *et al* developed a QSAR model on HIV-1 protease inhibitors using molecular connectivity index [29]. Gupta *et al* showed the efficiency of topological descriptors in predicting the anti-malarial activity [30]. In our previous study, we have shown the efficiency of molecular connectivity index and topological descriptor in determining the anti-Parkinson’s disease activity [31]. Further, Zhou *et al* showed the effect of constitutional, topological, and walk and path counts descriptors towards the antitumor activity [32]. Of 247 generated descriptors, 49 were shown to have no changes in descriptors values across the molecules in both the data sets, suggesting that these descriptors have no influence in classifying the groups as *“+HIA* versus *–HIA*” and “*+caco-2* versus *–caco-2″*, respectively. Hence, these 49 descriptors were eliminated from the *original data sets*, and the analyses were carried out against 198 descriptors (Figure 2). In addition, the *balanced data sets* were created from HIA and caco-2 *original data sets*, containing an equal number of positive and negative labeled instances (1∶1) for multivariate analysis.

### Multivariate Analysis

Partial least square discriminant analysis (PLS-DA) of HIA and caco-2 *balanced data sets* resulted in significant separation into two groups for each data set (p≤0.01 by permutation test; Figure 3A, B). The variable influence on the projection (VIP) parameter was used to select descriptors that showed significant contribution in discriminating the groups in PLS-DA models. Using the VIP cut-off value (VIP≥1), the number of descriptors discriminating against the HIA and caco-2 data sets were reduced to 49 and 50 descriptors, respectively (Figure 3C, D). The statistical analysis of these selected descriptors showed a significant differentiation (*p<0.05*) between their groups (Figure 3E, F). The results demonstrate that the selected descriptors are attributed as the most contributing descriptors in classifying each data set into groups. Furthermore, these descriptors were used to reconstruct the *original data sets* to determine the performance using machine learning algorithms (while other descriptors were eliminated from the original data sets).

### Selection of the Best Machine Learning Algorithm

The 21 machine learning classifiers have been trained and tested using the selected descriptors of *original data sets* (HIA and caco-2). The detailed performance of different classifiers on the *original data set* is shown in Figure 4A, B. Though the analysis involved two categories of data sets (HIA and caco-2), the logistic algorithm performs better in both the data sets compared with other classifiers. The logistic classifier showed an accuracy of 85.09 and 78.30% for HIA and caco-2 data set. The next two closest contending algorithms for HIA model are multilayer perceptron and random forest. Similarly, IB1 and IBK are the next two closest contending algorithms for caco-2 predictions. Overall, the performance of the logistic algorithm is extremely fast and more accurate compared to other analyzed classifiers.

To determine the efficiency of each descriptor in the logistic algorithm, we analyzed the individual descriptors that contributed to predictive accuracy on both the models. In HIA model, the combined accuracy of all 49 descriptors reached 85.09% whereas, the contribution of individual descriptors was significantly lower between 77–81% (Figure 4C). Similar trend was noticed in caco-2 model. The accuracy of each descriptor was lower, ranging between 59–70% (Figure 4D). Although, the individual contribution of each descriptor was significantly lower, the combined performance of descriptors enriched the accuracy in both the models. To estimate the optimal combination of these descriptors, a correlation based feature selection (CFS**)** [33], [34] algorithm was executed using weka software. Of PLS–DA optimized descriptors, nine from HIA and eight from caco-2 model were selected as an optimal subset using CFS analysis (Figure 5). However, the combined performance of these newly extracted descriptors decreases the accuracy of the logistic model in both the data sets (Figure 6A). Hence, further reduction of PLS-DA extracted descriptors will decrease the accuracy of the models with the newly extracted descriptors. Thus, PLS-DA based descriptors selections were confirmed to be an optimal set for the prediction of HIA and caco-2 permeability.

### Efficiency of Models against Smaller Data Sets

To ensure that the accuracy of the logistic algorithm is not only attributed to the larger data sets, the performance of the algorithm on smaller data set was calculated using the scoring algorithm. For instance, out of 1000 smaller HIA data sets, 680 data sets showed optimal accuracy compared to the accuracy of original HIA data set (Figure 6B). Therefore, the HIA score was calculated as 6.80. Similarly, the score for caco-2 smaller data sets were calculated as 7.10 (Figure 6C). This confirms that the selected descriptors of both the models are optimal and efficient in prediction of smaller data set.

### Integrative Analysis of Descriptors

Integrating the descriptors showed 47 are commonly associated to both the models (Figure 7). These 47 descriptors may be efficient in predicting the oral bioavailability data set (see Table S2, for the significance of the descriptors). In addition, the descriptors *RBN* and *TI2* were identified as unique to HIA model, whose predictive potentials were 77.6 and 78.2%, respectively. Similarly, *Jhete, Qindex* and *MAXDN* were specific to caco-2 data set and their predictive potentials were 62.2, 68.6 and 69.7%, respectively. Overall, these unique descriptors showed better accuracy in contributing to HIA and caco-2 prediction.

### Efficiency of Descriptors in Oral Bioavailability Prediction

To determine the best machine learning algorithm for the oral bioavailability prediction, the success rate of 21 classifiers using these 47 descriptors on bioavailability data set was compared. The best-performing algorithm was the logistic classifier, which is a regression model with a ridge estimator. The logistic classifier was able to classify the oral bioavailability data set with 71.19% accuracy (Figure 8A). The analysis of the descriptors showed a significant difference (*p<0.05*) between their groups (Figure 8B). Further, the performance of individual descriptors was analyzed from the set of 47 descriptors that contributed to predictive accuracy. Though, the combination of all 47 descriptors reached 71.19%, the predictive potential of most individual descriptors was significantly lower, between 50–60% (Figure 8A). Of these 47 descriptors, *GMTI, GMTIV, HyDp, QW, SMTI, SMTIV, TIE, TI1, RAM* and *ZM1V* are the most contributing descriptors with the accuracies approaching 70%.

To estimate the optimum combination of these 47 descriptors, we employed the CFS algorithm, using Weka software which extracts nine descriptors as an optimal subset (Figure 5). However, the combined performance of these nine descriptors decreases the accuracy of the model (Figure 6A). Hence, no further reduction of the descriptor set was possible, as the performance of logistic classifier dropped if any one of these 47 descriptors was eliminated. Since the bioavailability data set had 969 molecules, one may argue that the high performance of the logistic algorithm is a result of larger data set, rather than a generalization of the classifier. To confirm that the accuracy is not only attributed to the larger data sets, the scoring algorithm was executed for the small size of the data sets, as mentioned in methodology. The model showed to be fit with a score of 7.41 on smaller data sets (Figure 6D). Overall, the results suggest that these 47 descriptors are the fundamental for oral bioavailability predication, irrespective of the size of the data set. Interestingly, among these 47 descriptors, 39 were topological descriptors and remaining were associated to constitutional descriptors (Figure 2) suggesting that both these descriptors play a vital role in predicting oral bioavailability.

In conclusion, the systems biological approach was used on HIA and caco-2 data set to determine the major contributing descriptors for oral bioavailability prediction. Overall, 47 descriptors were identified as common between HIA and caco-2 permeability, and it is validated to be crucial in predicting oral bioavailability. Further, the predictions on small data sets demonstrate that this model holds good in estimating the oral bioavailability irrespective of data set size. Moreover, the logistic algorithm and the 47 selected attributes seem to capture the fundamental features of oral bioavailability and can predict oral bioavailability with an accuracy >71% for molecules with diverse structure. However, the accuracy of the model is limited, which suggest that the molecules have to meet a set of physicochemical requirements in addition to the analyzed descriptors. Hence, generating more valuable descriptors from other resources such as CDK, PEDAL, Power-MV may significantly improve the prediction limits. Overall, this study shows that the choices of both machine learning algorithm and optimal descriptor sets are critical for the prediction tasks. Conceivably, a similar approach can be used for the prediction of most contributing descriptors involved in drug toxicity.

## Materials and Methods

### Computational Framework

The systems biological framework (Figure 1) shows an overview of our method, which involves (i) compilation and curation of data sets, (ii) generating descriptors, (iii) multivariate analysis, (iv) selection of best-performing machine learning algorithm, (v) efficiency of the model in smaller data sets, and (vi) statistical analyses.

### Compilation and Curation of Dataset

The molecules associated with HIA, caco-2 permeability and oral bioavailability were derived from several literatures [8], [14], [20]–[28]. These structurally heterogeneous molecules were reported by different laboratories, which employed different experimental conditions and procedures to obtain biological property. Further, it was curated based on two-fold. *First,* if the molecules were reported in multiple articles, the average activity value was calculated. *Second*, the molecules were excluded, if there is a large discrepancy in activity value between the articles. Overall, 705, 605 and 969 molecules of intestinal absorption, caco-2 permeability and oral bioavailability (Table S1) were obtained, respectively.

### Generation Descriptors and Classification

The molecular descriptors representing the physicochemical properties of the molecules were derived using the E-dragon 1.0 version software [35]. The most frequently used descriptors [29]–[32] were analyzed, which include constitutional, connectivity indices, topological, and walk and path counts. To develop a reliable model, the descriptor space should be reduced by extracting most significant descriptors. Before the descriptors were reduced, the molecules in each data set were manually classified as positive and negative instance. For example, in oral bioavailability data set, the molecules with bioavailability (%F) values ≥80% were considered as “+bioavailability”, while the molecules with <80% were considered as “–bioavailability” [20]. Similar trends were followed for the molecules in HIA and caco-2 data set. The molecules with HIA (%FA) values ≥70% were considered as “+HIA” and the molecules with caco-2 permeability (logP_app_) values ≤–4 were considered as “+caco-2″, while remaining molecules were considered as “–HIA” and “–caco-2″, respectively [22]. Further, the balanced data sets were created from HIA and caco-2 data set by random selection to have an equal number of molecules with positive and negative instance for multivariate analysis.

### Multivariate Statistical Analysis

Partial least squares discriminant analysis (PLS-DA) was performed using SIMCA-P 12 (Umetrics AB, Umeå, Sweden) on the *balanced data sets* to extract the underlying descriptors, that discriminate between distinct classes (“+HIA versus –HIA” and “+caco-2 versus –caco-2″). The importance of each descriptor in the PLS-DA was evaluated by variable importance in the projection (VIP) scores. The VIP score positively reflects the descriptor's influence on the classification, and descriptors with a score greater than one were considered important in this study.

### Machine Learning

The classification learning algorithms used in this study were taken from the Weka software (Waikato Environment for Knowledge Analysis). The evaluation parameters were calculated with a ten times, ten-fold cross-evaluation. The method uses four-fifths of the data for training the model while the remaining fifth was used as a test set for estimating the performance based on the average value of accuracy and precision of ten-times validation.
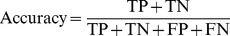


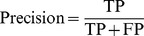
TP  =  True positive; TN  =  True negative; FP  =  False positive; FN  =  False negative.

### Efficiency of Model against Smaller Data Sets

To validate the efficiency of the models in smaller data sets, the scoring algorithm was evaluated as mentioned below,

1. Randomly split (with replacement) the molecules into 1000 smaller data sets, considering 100 molecules in each data set.
2. Compute the logistic algorithm with 10-fold cross-validation for each small data set.
3. Calculate the number of times, the accuracy of small data sets reach the equal and maximum accuracy of larger data set (*m*).
4. Finally, compute the *score*.

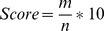



 ***m*** *:* number of times the small data sets showed equal and maximum accuracy as the accuracy of larger data set (e.g.: *bioavailability*  = 71.19%; *HIA*  = 85.09%; *caco-2* = 78.30%).***n***: Total number of small data sets (1000).***Significance level*** *:* score ≥5.

### Statistical Analysis

The significance of descriptors was estimated by the analysis of variance (ANOVA), using GeneSpring GX7.3 microarray software (Agilent Technologies Inc., Santa Clara, California) to make comparisons between positive and negative instances. A total of three comparisons were made: *+HIA* versus the *–HIA*, *+caco-2* versus *–caco-2*, and the *+bioavailability* versus *– bioavailability*. The *p-value* ≤0.05 were regarded as significance.

## Supporting Information

Table S1 **Curated Data sets.**
(XLS)

Table S2 **Significance of the analyzed descriptors.**
(XLS)

## References

[1] Ahmed SS, Ahameethunisa AR, Santosh W, Chakravarthy S, Kumar S (2011). Systems biological approach on neurological disorders: a novel molecular connectivity to aging and psychiatric diseases.. BMC Syst Biol.

[2] Li J, Zhu X, Chen JY (2009). Building disease-specific drug-protein connectivity maps from molecular interaction networks and PubMed abstracts.. PLoS Comput Biol.

[3] Hu G, Agarwal P (2009). Human disease-drug network based on genomic expression Profiles.. PLoS ONE.

[4] Graham RJ, Robert ZH, David TL (2001). Pharmacokinetics and Its Role in Small Molecule Drug Discovery Research.. Med Res Rev.

[5] Nassar AE, Kamel AM, Clarimont C (2004). Improving the decision-making process in the structural modification of drug candidates: enhancing metabolic stabilit Drug Discov Today.

[6] Kennedy T (1997). Managing the drug discovery/development interface.. Drug Discov Today.

[7] Caldwell GW (2000). Compound optimization in early- and late-phase drug discovery: acceptable pharmacokinetic properties utilizing combined physicochemical, in vitro and in vivo screens.. Curr Opin Drug Discov Devel.

[8] Hou T, Wang J, Zhang W, Xu X (2007). ADME evaluation in drug discovery. 6. Can oral bioavailability in humans be effectively predicted by simple molecular property-based rules?. J Chem Inf Model.

[9] Ruiz-Garcia A, Bermejo M, M Hu, X Li (2011). *In vivo* Methods for Oral Bioavailability Studies..

[10] Hou T, Wang J, Zhang W, Xu X (2007). ADME evaluation in drug discovery. 7. Prediction of oral absorption by correlation and classification.. J Chem Inf Model.

[11] Andrews CW, Bennett L, Yu LX (2000). Predicting human oral bioavailability of a compound: development of a novel quantitative structure-bioavailability relationship.. Pharm Res.

[12] Yoshida F, Topliss JG (2000). QSAR model for drug human oral bioavailability.. J Med Chem.

[13] Agatonovic-Kustrin S (2003). Prediction of drug bioavailability based on molecular structure. Anal.. Chim.Acta.

[14] Veber DF, Johnson SR, Cheng HY, Smith BR, Ward KW (2002). Molecular properties that influence the oral bioavailability of drug candidates.. J Med Chem.

[15] Wang JM, Krudy G, Xie XQ, Wu CD, Holland G (2006). Genetic algorithm-optimized QSPR models for bioavailability, protein binding, and urinary excretion. J. Chem. Inf.. Model.

[16] Ma CY, Yang SY, Zhang H, Xiang ML, Huang Q, Wei YQ (2008). Prediction models of human plasma protein binding rate and oral bioavailability derived by using GA-CG-SVM method. J.Pharmaceut.. Biomed.

[17] Tian S, Li Y, Wang J, Zhang J, Hou T (2011). ADME evaluation in drug discovery. 9. Prediction of oral bioavailability in humans based on molecular properties and structural fingerprints.. Mol Pharm.

[18] Zhu J, Wang J, Yu H, Li Y, Hou T (2011). Recent developments of in silico predictions of oral bioavailability.. Comb Chem High Throughput Screen.

[19] Han V, Bernard T (1998). Drug Bioavailability: Estimation of solubility, permeability, absorption and bioavailability.. Wiley-vch, weinheim.

[20] Moda TL, Montanari CA, Andricopulo AD (2007). Hologram QSAR model for the prediction of human oral bioavailability.. Bioorg Med Chem.

[21] Varma MV, Obach RS, Rotter C, Miller HR, Chang G (2010). Physicochemical space for optimum oral bioavailability: contribution of human intestinal absorption and first-pass elimination.. J Med Chem.

[22] Subramanian G, Kitchen DB (2006). Computational approaches for modeling human intestinal absorption and permeability.. J Mol Model.

[23] Yan A, Wang Z, Cai Z (2008). Prediction of human intestinal absorption by GA feature selection and support vector machine regression.. Int J Mol Sci.

[24] Hou T, Wang J, Li Y (2007). ADME evaluation in drug discovery. 8. The prediction of human intestinal absorption by a support vector machine.. J Chem Inf Model.

[25] Hai PT, Isabel G, Marival B, Victor MS, Inmaculada C (2011). *In Silico* prediction of caco-2 cell permeability by a classification QSAR approach.. Mol Inform.

[26] Paixão P, Gouveia LF, Morais JA (2010). Prediction of the in vitro permeability determined in Caco-2 cells by using artificial neural networks.. Eur J Pharm Sci.

[27] Hou TJ, Zhang W, Xia K, Qiao XB, Xu XJ (2004). ADME evaluation in drug discovery. 5. Correlation of Caco-2 permeation with simple molecular properties.. J Chem Inf Comput Sci.

[28] Fujiwara S, Yamashita F, Hashida M (2002). Prediction of Caco-2 cell permeability using a combination of MO-calculation and neural network. Int J Pharm..

[29] Gayathri P, Pande V, Sivakumar R, Gupta SP (2001). A quantitative structure-activity relationship study on some HIV-1 protease inhibitors using molecular connectivity index.. Bioorg Med Chem.

[30] Gupta MK, Prabhakar YS (2006). Topological descriptors in modeling the antimalarial activity of 4-(3',5'-disubstituted anilino) quinolines.. J Chem Inf Model.

[31] Ahmed SS, Ahameethunisa A, Santosh W (2010). QSAR and pharmacophore modeling of 4-arylthieno [3, 2-d] pyrimidine derivatives against adenosine receptor of Parkinson’s disease.. J Theor Comput Chem.

[32] Zhou W, Dai Z, Chen Y, Wang H, Yuan Z (2012). High-dimensional descriptor selection and computational qsar modeling for antitumor activity of arc-111 analogues based on support vector regression (SVR).. Int J Mol Sci.

[33] Ooi CH, Chetty M, Teng SW (2006). Differential prioritization between relevance and redundancy in correlation-based feature selection techniques for multiclass gene expression data.. BMC Bioinformatics.

[34] Reddy AS, Kumar S, Garg R (2010). Hybrid-genetic algorithm based descriptor optimization and QSAR models for predicting the biological activity of Tipranavir analogs for HIV protease inhibition.. J Mol Graph Model.

[35] Tetko IV, Gasteiger J, Todeschini R, Mauri A, Livingstone D (2005). Virtual computational chemistry laboratory–design and description.. J Comput Aided Mol Des.

